# Infection by the Helminth Parasite *Fasciola hepatica* Requires Rapid Regulation of Metabolic, Virulence, and Invasive Factors to Adjust to Its Mammalian Host*[Fn FN2]

**DOI:** 10.1074/mcp.RA117.000445

**Published:** 2018-01-10

**Authors:** Krystyna Cwiklinski, Heather Jewhurst, Paul McVeigh, Tara Barbour, Aaron G. Maule, Jose Tort, Sandra M. O'Neill, Mark W. Robinson, Sheila Donnelly, John P. Dalton

**Affiliations:** From the ‡School of Biological Sciences, Medical Biology Centre, Queen's University Belfast, Belfast, Northern Ireland, UK;; §Institute for Global Food Security (IGFS), Queen's University Belfast, Belfast, Northern Ireland, UK;; ¶Departamento de Genética, Facultad de Medicina, Universidad de la República, Uruguay;; ‖School of Biotechnology, Dublin City University, Ireland;; **The i3 Institute and School of Medical and Molecular Biosciences, University of Technology, Sydney, Australia

## Abstract

The parasite *Fasciola hepatica* infects a broad range of mammals with impunity. Following ingestion of parasites (metacercariae) by the host, newly excysted juveniles (NEJ) emerge from their cysts, rapidly penetrate the duodenal wall and migrate to the liver. Successful infection takes just a few hours and involves negotiating hurdles presented by host macromolecules, tissues and micro-environments, as well as the immune system. Here, transcriptome and proteome analysis of *ex vivo F. hepatica* metacercariae and NEJ reveal the rapidity and multitude of metabolic and developmental alterations that take place in order for the parasite to establish infection. We found that metacercariae despite being encased in a cyst are metabolically active, and primed for infection. Following excystment, NEJ expend vital energy stores and rapidly adjust their metabolic pathways to cope with their new and increasingly anaerobic environment. Temperature increases induce neoblast proliferation and the remarkable up-regulation of genes associated with growth and development. Cysteine proteases synthesized by gastrodermal cells are secreted to facilitate invasion and tissue degradation, and tegumental transporters, such as aquaporins, are varied to deal with osmotic/salinity changes. Major proteins of the total NEJ secretome include proteases, protease inhibitors and anti-oxidants, and an array of immunomodulators that likely disarm host innate immune effector cells. Thus, the challenges of infection by *F. hepatica* parasites are met by rapid metabolic and physiological adjustments that expedite tissue invasion and immune evasion; these changes facilitate parasite growth, development and maturation. Our molecular analysis of the critical processes involved in host invasion has identified key targets for future drug and vaccine strategies directed at preventing parasite infection.

The helminth parasite, *Fasciola hepatica*, is an economically important pathogen of livestock worldwide, and an increasingly reported zoonotic pathogen in Asia, Africa and South America (1–4). Infection of the mammalian host by *F. hepatica* occurs following the ingestion of vegetation contaminated with the encysted stage, the metacercariae. The double-layered cyst protects the parasite on pasture from changing ambient temperatures and precipitation (5). Acid proteases within the stomach or rumen remove the outer layer while reducing conditions, bile salts, CO_2_ tension and neutral pH within the duodenum induce the parasites to emerge from the inner cyst as newly excysted juveniles (NEJ)^1^. These rapidly traverse the intestinal wall and migrate to the liver. Within the liver, the juveniles move through the parenchyma tissue to the bile ducts where they develop into sexually mature adults (5, 6).

During these early infection and migration processes the parasite encounters different tissues, varying micro-environments, and host innate immune cells that are alerted by parasite molecules. However, histological and immunological studies have shown that the intestinal wall offers little resistance to invasion by NEJ and that the parasites can quickly manipulate the host's immune response. Within hours, the parasites prevent the onset of protective Th1-mediated immune responses by modulating protective innate cells, such as macrophages, to prime Th2 responses that benefit their survival (7, 8). Remarkably, *F. hepatica* can infect a wider range of terrestrial mammals than any other helminth parasite (3), ranging from rodents, lagomorphs, ungulates, ruminants, marsupials, camelids and primates. The parasite first encountered several of these mammalian hosts, such as kangaroos, coypus and camelids, in very recent times (< 400 years ago), suggesting that they have evolved very effective and universal processes of invasion, virulence and immune modulation (3).

We recently reported the sequencing of the *F. hepatica* genome from a UK isolate (9), which was found to be among the largest helminth genomes at 1.3Gb and highly polymorphic. Further genome sequencing by McNulty and colleagues (10) revealed that *F. hepatica* isolates from the Americas were colonized with *Neorickettsia* endobacteria; whether or not this endobacteria and *F. hepatica* have a endosymbiotic relationship similar to *Wolbachia* and filarial nematodes (11), has yet to be determined. In both genome data sets, many genes, for example those encoding cysteine proteases, have expanded and diverged to create families of proteins with overlapping but broad functions. These features likely contribute to the high adaptability of the parasite to different hosts, to their successful global expansion as well as their ability to produce drug resistant isolates. Indeed, over the last three decades the spread of parasites resistant to one of the most effective anti-*Fasciola* drugs, triclabendazole, has left farming communities with limited options for effective fluke control (12, 13) and may be contributing to increased prevalence of fascioliosis, at least in Europe (14). Moreover, because triclabendazole is the only licensed drug for human fasciolosis the emergence of resistant parasites has significant future medical implications (15, 16). The development of new means of combatting fasciolosis, either by chemical treatment or vaccination, is imperative.

Despite the extensive pathology caused by the metacercariae and NEJ stages of *F. hepatica* in human and animal fasciolosis, there is a dearth of information on their biology, largely because of their microscopic size and difficulties associated with laboratory propagation. Supported by the availability of the parasite's genome (9), we have now performed an in-depth transcriptomic and proteomic study focused on understanding the key metabolic, biochemical and molecular mechanisms underpinning parasite excystment, invasion, virulence and development in the first 24 h postexcystment, at a time when the parasite must contend with many host-related obstacles. Our data reveal a parasite prepared to “run the gauntlet,” with an ability to quickly up-regulate many genes in response to changes in its environment that facilitate tissue penetration and invasion of the host. Simultaneously, these alterations enable development, including the proliferation of neoblast-like stem cells, consistent with the accelerated growth the parasite undergoes when established within the mammalian host. Analysis of the somatic and secretory proteome was compared with gene expression and unveiled a diverse range of early-stage proteins required for virulence and modulation of the host immune response which represent key targets for future drug and vaccine development.

## EXPERIMENTAL PROCEDURES

### 

#### 

##### Experimental Design and Statistical Rationale

RNAseq and proteomic analysis of the *ex vivo* early infective stage parasites was carried as illustrated by supplemental Fig. S1. Specifically, RNAseq analysis was carried out on biological replicates (3000–3500 parasites/replicate) of metacercariae (3 replicates), NEJ 1 h post excystment (2 replicates), NEJ 3 h post excystment (2 replicates), and NEJ 24 h post excystment (2 replicates). Protein samples from biological replicates were used for proteomic analysis of both the secreted and somatic proteins as follows; (a) secretome analysis (3000–3500 parasites/replicate): NEJ 1 h post-excystment (4 replicates), NEJ 3 h post-excystment (3 replicates), and NEJ 24 h post-excystment (3 replicates); (b) somatic proteome analysis (1000 parasites/replicate): metacercariae (3 replicates, NEJ 3 h post-excystment (3 replicates), NEJ 24 h post-excystment (3 replicates), and NEJ 48 h post-excystment (2 replicates). Proteomic analysis was based on protein identification with at least two unique peptides in at least two of the biological replicates to determine relevant protein identification and quantification. Subsequent analysis was carried out on mean values for each developmental time point with at least a mean of two unique peptides being identified. qPCR analysis was carried out in triplicate, including no template negative controls and analyzed using One Way ANOVA with Tukey's post hoc tests according to standard protocols. Statistical differences in the number of neoblasts detected under different experimental conditions were analyzed using One Way ANOVA with Tukey's post hoc tests according to standard protocols. The results of the whole NEJ immunolocalization were reflective of at least 20 individual NEJ analyzed for each time point.

##### Source of Parasite Material

A North American isolate was used for the analysis of the parasite transcriptomes and secretomes carried out within this study, sourced from Baldwin Aquatics Inc. (Monmouth, OR, United States). Ridgeway Research Ltd. (St Briavels, UK) supplied the metacercariae; South Gloucester isolate for the somatic proteome analysis and the Italian isolate for the neoblast analysis and cathepsin cysteine protease immunolocalization and qPCR analysis.

##### Metacercariae Excystment and Parasite Culture

Metacercariae were incubated for a maximum of 10 min in 2% sodium hypochlorite with agitation at room temperature to remove the outer cyst wall. The parasites were washed in distilled water by sedimentation to remove all traces of sodium hypochlorite. The washed parasites were resuspended in excystment medium (1.2% sodium bicarbonate, 0.9% sodium chloride, 0.2% sodium tauroglycocholate, 0.07% concentrated hydrochloric acid, 0.006% l-cysteine) and incubated for up to 3 h at 37 °C in 5% CO_2_.

NEJ were recovered using a pipette at 1 h and 3 h. Further NEJ were incubated for 24 h in prewarmed (37 °C) culture medium (RPMI 1640 medium (ThermoFisher Scientific, Waltham, MA) containing 2 mm l-glutamine, 30 mm HEPES, 0.1% (w/v) glucose, and 2.5 μg/ml gentamycin) followed by centrifugation at 400 × *g*. The NEJ pellet was washed three times with PBS and stored at −80 °C prior to RNA extraction. The supernatant, the excretory-secretory (ES) protein fraction, of each of these stages was recovered for proteomic analysis.

##### Transcriptome Sequencing (RNASeq)

Total RNA was extracted using TRIzol (ThermoFisher Scientific) according to the manufacturer's instructions. RNA integrity and concentration were confirmed using the Bioanalyzer 2100 (Agilent Technologies, Stockport, UK) and Nanodrop, respectively. llumina TruSeq RNA libraries (nonstranded) were prepared with 4 μg of total RNA taken from biological replicates of metacercariae, NEJ 1 h post excystment, NEJ 3 h post excystment, NEJ 24 h post excystment at Genome Quebec (Montreal, Canada) and sequenced (Pair-end 100bp) on a HiSEQ 2500 (Illumina), resulting in at least 66 million reads per sample.

##### Assembly, Annotation, and Gene Expression Analysis

Illumina HiSeq reads were trimmed to Q≥30 and adaptors removed using Fastx_toolkit (version 0.0.13). RNAseq libraries were mapped to the putatively annotated *F. hepatica* MAKER gene models (9) using TopHat2 (17) and read counts extracted using htseq-count. Based on these counts normalized transcript abundance was calculated as transcripts per million (TPM), with subsequent analysis carried out on those genes with a normalized count of at least two TPM. Comparison of the number of genes transcribed by each time point were visualized using an Upset Plot (18) (supplemental Fig. S2). Comparative analysis with the transcriptomic responses of juvenile 21 day old and adult parasites was carried out on RNAseq data generated from samples isolated from rats and bovine infected with *F. hepatica* as detailed by Cwiklinski *et al.* (9).

Network analysis of the 17901 genes expressed within the first 24 h post-excystment was carried using a Network graph constructed using BioLayout Express^3D^ (19) with a Pearson correlation threshold of r ≥ 0.97. The graph comprised of 13,559 nodes connected by 765,001 edges, which was clustered using the Markov clustering algorithm (MCL 2.2.6), resulting in 857 clusters with at least 4 nodes that were temporally expressed by the different lifecycle stages; metacercariae, NEJ 1 h, 3 h, and 24 h post-excystment. Hierarchical clustering was also carried out on those genes that displayed at least a 2-fold difference in expression among any of the four lifecycle stages, represented by 6009 genes with a baseline cut-off of 2 TPM, graphically represented using heatmaps generated using the R program, pheatmap. Gene model annotation was carried out using Uniprot, Gene Ontology and Interpro *in silico* tools (9) and the KEGG Automatic Annotation Server (KAAS; (20). Metabolic pathway analysis was carried by normalizing the global patterns of expression at the KEGG module level (21, 22); graphically represented using heatmaps generated using the R program, pheatmap.

##### Liquid Chromatography and Tandem Mass Spectrometry (LC-MS/MS)

Protein digestion and mass spectrometry analyses were carried out by the Proteomics Platform of the Quebec Genomics Center (CHU de Quebec Research Centre, Laval, Canada). Secreted proteins (10 μg) were concentrated 10 times from biological replicates of NEJ 1 h post-excystment, NEJ 3 h post-excystment and NEJ 24 h post-excystment, followed by 3 washes using an Amicon Ultra 3kDa column with 50 mm ammonium bicarbonate buffer before being dried by evaporation in a SpeedVac (ThermoFisher Scientific). Somatic proteins were extracted from biological replicates of metacercariae and NEJ parasites at 3 h, 24 h and 48 h post-excystment excysted as above, by homogenization in RIPA buffer (50 mm Tris-HCl, pH 7.2, 150 mm NaCl, 1 mm Pefabloc (Sigma-Aldrich, Dorset, UK), 1 mm EDTA, 1% Triton X-100, 1% sodium deoxycholate (Sigma-Aldrich), 0.1% SDS, 1 mm E64 (Sigma-Aldrich) and placed on ice for 30 min. The extracted proteins were then centrifuged at 13,000 × *g* for 10 min to remove any insoluble components and the supernatant stored at −20 °C until use. Proteins were analyzed by 1-DE SDS-PAGE using 4–12% Criterion XT gels (BioRad, Mississauga, Canada) and staining with SYPRO Ruby protein gel stain (ThermoFisher Scientific). Bands of interest were extracted from gels and placed in 96-well plates and washed with water.

In-gel tryptic digestion was performed on a MassPrep liquid handling robot (Waters, Milford, USA) according to the manufacturer's specifications and to the protocol of Shevchenko *et al.* (23) with the modifications suggested by Havlis *et al.* (24). Briefly, proteins were reduced with 10 mm DTT and alkylated with 55 mm iodoacetamide. Trypsin digestion was performed using 126 nm of modified porcine trypsin (Sequencing grade, Promega, Madison, WI) at 37 °C for 18 h. Digestion products were extracted using 1% formic acid, 2% acetonitrile followed by 1% formic acid, 50% acetonitrile. The recovered extracts were pooled, vacuum centrifuge dried and then resuspended into 10 μl of 0.1% formic acid. Mass spectrometry analysis was performed on a TripleTOF 5600 mass spectrometer fitted with a nanospray III ion source (ABSciex, Concord, ON) and coupled to an Agilent 1200 HPLC, using 2 μl of the resuspended sample.

##### Database Searching and Criteria for Protein Identification

MS/MS peak lists (MGF files) were generated using Paragon and Progroup algorithms (Protein Pilot version 4.5; ABSciex; (25) and analyzed using Mascot (version 2.4.1; Matrix Science) and X!Tandem (version CYCLONE; 2010.12.01.1). The secretome proteome data were set up to search against three custom *F. hepatica* databases, assuming digestion with trypsin with two missed cleavages permitted: (1) Database comprised of the gene models identified from the *F. hepatica* genome (v1; 101,780; (9), (2) Database comprised of all available *F. hepatica* EST sequences from NCBI and *F. hepatica* transcriptome sequencing projects (633,678 entries; ftp://ftp.sanger.ac.uk/pub/pathogens/Fasciola/; (26), (3) Database comprised of the Trematoda specific sequences within the nonredundant NCBI data set (1,541,675 entries). The protein identifications were consistent across all three databases, although the database derived from the draft *F. hepatica* genome resulted in the greatest number of protein identifications and thus were used for all subsequent analyses (supplemental Table S1). The somatic proteome data were set up to search against the *F. hepatica* database comprised of the gene models identified from the *F. hepatica* genome (101,780; (9). Fragment and parent ion mass tolerance were set at 0.100 Da. Carbamidomethylation of cysteine was specified as a fixed modification. Oxidation of methionine, deamidation of asparagine and glutamine and pyro glutamate formation of the N terminus (Glu->pyro-Glu and Gln->pyro-Glu) were specified as variable modifications. Scaffold (v4.3.2; Proteome Software Inc, Portland, OR) was used to validate MS/MS based peptide and protein identifications and calculate protein abundance using the Exponentially Modified Protein Abundance Index (emPAI). Peptide identifications were accepted if they could be established at greater than 95% probability by the Peptide Prophet algorithm (27) with Scaffold delta-mass correction to achieve an FDR less than 1% by the Scaffold Local FDR algorithm (27). Protein identifications were accepted if they could be established at greater than 95% probability to achieve an FDR less than 1% and contained at least 2 identified peptides. Protein probabilities were assigned by the Protein Prophet algorithm (28). Proteins that contained similar peptides and could not be differentiated based on MS/MS analysis alone were grouped to satisfy the principles of parsimony. Spearman correlation analysis determined the reproducibility of biological replicates, with all developmental time-points sharing a significant positive correlation. Differences in protein abundance based on mean emPAI values among the developmental time-points were graphically represented by heat maps.

##### Whole NEJ Immunolocalization by Confocal Microcopy

*F. hepatica* metacercariae were excysted as described above and NEJ cultured in RPMI 1640 medium containing 2 mm l-glutamine, 30 mm HEPES, 0.1% (w/v) glucose, 2.5 μg/ml gentamycin and 10% fetal calf serum (ThermoFisher Scientific) for up to 48 h. NEJ were removed directly after excystment, and then following 1 h, 6 h, 10 h, 24 h, and 48 h of culture in RPMI 1640 medium and fixed for immunolocalization studies. A subset of the NEJ was also stored for RNA extraction for validation of the protease transcripts by qPCR.

The parasites were fixed with 4% paraformaldehyde in 0.1 m PBS (Sigma-Aldrich) for 1 h at room temperature and then washed three times with antibody diluent (AbD: 0.1 m PBS containing 0.1% (V/V) Triton X-100, 0.1% (W/V) bovine serum albumin and 0.1% (W/V) sodium azide). NEJ were then incubated in AbD containing either anti-FhCL3 antiserum (prepared in rabbit against recombinant FhCL3) at a 1:500 dilution or anti-FhCB antiserum (prepared in rabbit against recombinant FhCB2) at a 1:500 dilution, overnight at 4 °C, followed by three washes in AbD. As a negative control, separate samples were incubated in AbD containing rabbit preimmune antiserum at a 1:500 dilution. Following washing, all NEJ samples were incubated in a 1:200 dilution of the secondary antibody, fluorescein isothiocyanate (FITC)-labeled goat anti-rabbit IgG (Sigma-Aldrich) in AbD overnight at 4 °C, followed by three washes in AbD. To counter-stain muscle tissues, NEJ were incubated in AbD containing 200 μg/ml phalloidin conjugated to tetramethylrhodamine isothiocyanate (TRITC) overnight at 4 °C. Following three final washes in AbD, NEJ were whole-mounted in a 9:1 glycerol solution containing 0.1 m propyl gallate and viewed using confocal scanning laser microscopy (CSLM) (Leica TCS SP8) under the HCX PL APO CS 100× oil objective lens. Leica type F immersion oil was used in viewing and all images taken at room temperature.

##### Neoblast Labeling and Visualization

Metacercariae were excysted and cultured in RPMI 1640 medium, as previously described with the addition to 10% Fetal calf serum (FCS). 5-ethynyl-2-deoxyuridine (EdU) labeling and detection was carried out using the Click-iT^®^ EdU Imaging Kit (ThermoFisher Scientific), performed in triplicate per time point. NEJ were cultured in the presence of 10 μm EdU for 24 h. Following the EdU pulse, the NEJ were fixed for 30 min at room temperature in 4% formaldehyde in PBS with 0.2% Triton X-100. The fixed parasites were sequentially dehydrated in 50% methanol and then 100% methanol, followed by an overnight incubation at −20 °C. The samples were rehydrated by exchanging 100% methanol with 50% methanol and then PBSTx (PBS with 0.3% Triton X-100). EdU incorporation was detected by click reaction with Alexa Fluor azide (Click-iT^®^ EdU Imaging Kit; ThermoFisher Scientific) for 20 min, according to Wang *et al.* (29). NEJ were whole-mounted in a 9:1 glycerol solution containing 0.1 m propyl gallate and viewed using confocal scanning laser microscopy (CSLM) (Leica TCS SP8) under the HCX PL APO CS 100× oil objective lens. Leica type F immersion oil was used in viewing and all images taken at room temperature. Neoblast counts were carried out throughout all planes of view to identify neoblast-like cells throughout the NEJ. Statistical analysis was carried out by One Way ANOVA (version 6.00 for Windows, GraphPad Software); *p* value <0.05 was deemed statistically significant.

##### Expression of Neoblast-Associated Genes

Genes associated with neoblast-like stem cells inferred from the literature (29), were used to interrogate the *F. hepatica* genome and available transcriptome data. Differential gene expression analysis across the *F. hepatica* lifecycle was carried out for genes representing *histone 2a/2b*, *nanos*, *enhancer of zeste*, *tudor*, *argonaute 2*, and *vasa-like* genes (supplemental Table S2). To confirm transcription during the early stages of infection, a panel of five genes (*nanos*, *ago 2.1*, *ago 2.2*, *his 2a*, and *his 2b*) was validated by qPCR.

##### Quantitative Gene Expression Analysis (qPCR)

Total RNA was extracted from 100 NEJ per time point using the miRNeasy Mini Kit (Qiagen, Manchester, UK) according to the manufacturer's instructions, eluted in 30 μl RNase-free water. Assessment of RNA concentration and quality was carried out using the LVis plate functionality on the PolarStar Omega Spectrophotometer (BMG LabTech, Aylesbury, UK). cDNA synthesis was carried out using the High capacity cDNA reverse transcription kit (ThermoFisher Scientific) according to manufacturer's instructions. qPCR reactions were performed in 20 μl reaction volumes in triplicate, using 1 μl cDNA diluted 1:2, 10 μl of Platinum® SYBR® Green qPCR SuperMix-UDG kit (ThermoFisher Scientific) and 1 μm of each primer (supplemental Table S3). A negative control (no template) was included in each assay. qPCR was performed using a Rotor-Gene thermocycler (Qiagen), with the following cycling conditions: (1) neoblast study: 95 °C: 10 min; 40 cycles: 95 °C:10 s, annealing temperature:15 s, 72 °C: 20 s; 72 °C: 5 min, (2) cathepsin protease expression study: 95 °C: 10 min; 40 cycles: 95 °C:10 s, annealing temperature:30 s, 72 °C: 20 s; 72 °C: 5 min. Relative expression analysis was performed manually using Pfaffl's Augmented ΔΔCt method (30) whereby the comparative cycle threshold (Ct) values of the samples of interest are compared with a control and normalized to the housekeeping gene, Glyceraldehyde 3-phosphate dehydrogenase (GAPDH; AY005475). For this method to be valid, amplification efficiencies of individual reactions were verified using the comparative quantification package within the Rotor-Gene Q software v2.1.0. Annealing temperatures and melt-curve analysis was also carried out to check for single DNA products produced by these primer sets. Results were analyzed using One Way ANOVA (version 6.00 for Windows, GraphPad Software); *p* value <0.05 was deemed statistically significant.

## RESULTS AND DISCUSSION

### 

#### 

##### Rapid Regulation of Gene Expression in the First 24 h Post-Excystment is Critical to Establishing Infection

**Fig. 1.**
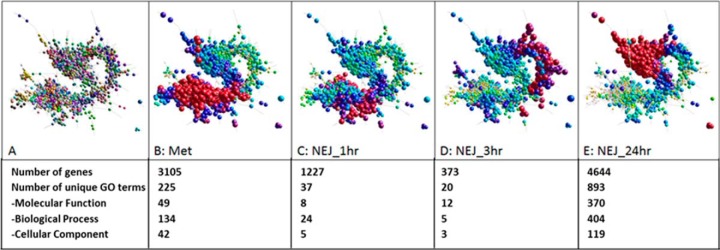
**Network graph of the 17901 genes expressed within the first 24 h.** *A*, 3D layout graph represented by 13,559 nodes connected by 765,001 edges at a Pearson correlation threshold of r ≥ 0.97. *B–E*, Temporal gene expression by the *F. hepatica* life-cycle stages: metacercariae (met), NEJ 1 h, 3 h, and 24 h post-excystment, respectively. Low levels of gene transcription are depicted by the small node size and the yellow/green node color. Increased gene transcription is represented by an increase in node size and the node color change from yellow/green to blue/purple/red. The number of genes with increased gene transcription at each time point and the number of unique Gene Ontology (GO) terms represented by these genes are shown.

By analyzing gene transcription within the first 24 h following excystment we identified a 17901 gene subset of the 22,677 gene models identified in the draft *F. hepatica* genome (9) that are transcribed and strictly regulated by the metacercariae and NEJ at 1 h, 3 h and 24 h post-excystment (Fig. 1; supplemental Fig. S2; supplemental Table S4; supplemental Table S5). Network analysis of these genes, based on a correlation threshold of r≥0.97 (Biolayout), revealed a pattern of temporal gene expression. As expected, given the short time intervals between the metacercariae and NEJ 1 h post-excystment, the NEJ 1 h transcribe a similar cohort of genes to the metacercariae (97% overlap; Fig. 1*B*–1*C*). By contrast, distinct clusters of regulated genes are observed in the NEJ 3 h (Fig. 1*D*) and NEJ 24 h (Fig. 1*E*) post-excystment. In total, 857 clusters with at least 4 nodes were generated, with the largest cluster of genes (Cluster 1: 2200 genes) expressed by the NEJ 24 h. This represents an increase in overall expression of a large number of genes, as shown by the 893 gene ontology terms uniquely identified in this cluster, which particularly encompass terms associated with metabolism, the cell cycle and growth (supplemental Table S4; supplemental Table S5).

**Fig. 2.**
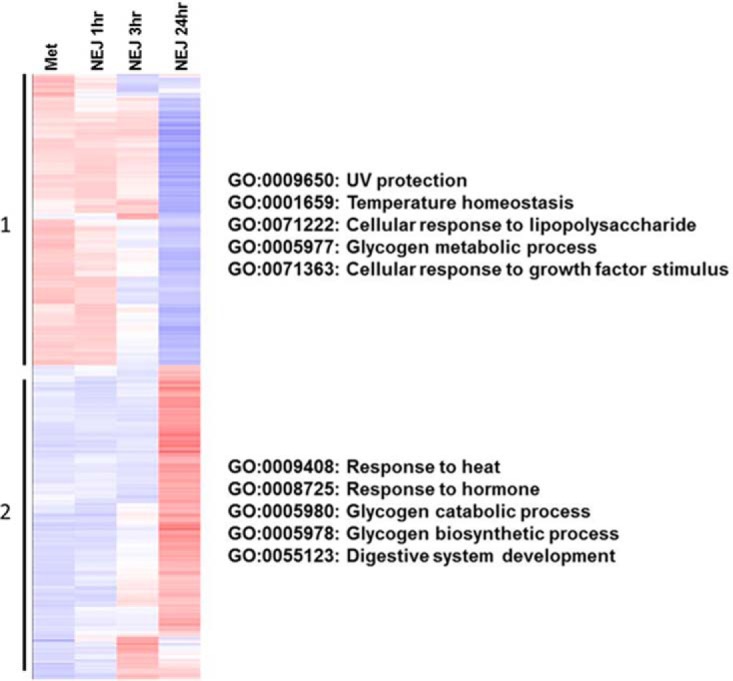
**Differential gene expression during the first 24 h.** Genes expressed by biological replicates of metacercariae and NEJ 1 h, 3 h and 24 h post-excystment with a baseline cut-off of 2 TPM were grouped by hierarchical clustering, represented by a heatmap (Up-regulation represented in red; down-regulation represented in blue). The 6009 gene models broadly clustered into two groups: (1) Genes up-regulated during the metacercariae and NEJ 1 h stages; (2) Genes showing an up-regulation during the NEJ 3 h and 24 h stages. Gene ontology terms reflecting biological processes associated with each group are shown.

Focused hierarchical clustering of genes that display at least a 2-fold difference in expression between any of the four lifecycle stages (metacercariae, NEJ 1 h, 3 h and 24 h) were broadly separated into two groups, consistent with the network analysis (Fig. 2). Gene ontology analysis of these two groups revealed unique GO terms that associated with three aspects of the parasite lifecycle relevant to establishing infection as follows:

1. The change from external environment to the mammalian host entailing increased temperature and salinity. For example, genes associated with UV protection and temperature homeostasis were upregulated in the metacercariae, whereas the upregulation of genes associated with the response to heat and hormones was observed in the NEJ 24 h.
2. Alterations to metabolism. Genes related to the metabolism of glycogen were more highly expressed by the metacercariae and NEJ 1 h compared with the genes associated with glycogen catabolism and synthesis which, conversely, were elevated in the NEJ 24 h. This observation is consistent with the free-living metacercariae being reliant on endogenous glycogen stores as a source of energy.
3. Growth and development. Within three hours of excystment the parasite expresses genes that are associated with the GO term GO:0071363 cellular response to growth factor stimulus. At 24 h, genes associated with development are also observed to be up-regulated, particularly genes linked with the development of the digestive system (GO:0055123), suggesting that the parasite changes from relying on its own glycogen stores to feeding on host tissues and blood.

Of the 6009 genes that display at least a 2-fold change between the metacercariae and the NEJ, several gene families were identified with regulated expression across this 24 h period. These genes included an array of heat shock proteins (HSP), including HSP-40, HSP-70, HSP-90 and small HSPs such as HSP-10 and alpha crystallin-containing small heat shock proteins, that are more highly expressed during the metacercariae and NEJ 1 h stages compared with the NEJ 3 h and 24 h stages. Studies of other helminths, including the *Schistosoma mansoni* schistosomula, *Echinococcus granulosus* larval stages and *Caenorhabditis elegans* dauer stage have shown that HSPs and redox based antioxidant enzymes are among the most highly expressed genes of the “dormant” and/or encysted stages (31–35), suggesting that they are essential in the response to sudden environmental changes that the parasite must endure when invading its host.

Intriguingly, *Fasciola* also expresses and temporally regulates several members of a family of aquaporins (aqp; water channels) within the first 24 h. As well as facilitating the transport of water, the aquaporin family consists of channels that facilitate the transport of glycerol, urea and other small solutes, termed aquaglyceroporins (36). The selectivity of the aquaporin channels is determined by two NPA motifs present within the transmembrane domains (36), an aromatic/arginine selectivity filter characterized by four amino acids (36, 37) and five conserved amino acid residues known as the Froger's residues (38). Analysis of the *F. hepatica* genome has revealed eight aquaporin-like genes, seven of which are transcribed by the metacercariae and NEJ (supplemental Fig. S3). Based on the above classification, *F. hepatica* expresses genes corresponding to both water transporting aquaporins (mammalian classification: *aqp-1* and *aqp-2*) and aquaglyceroporins (mammalian classification: *aqp-3* and *aqp-9*). Analysis of the expression of these genes shows that the water transporting aquaporin-like genes (*aqp-1-like* and *aqp-2-like*), are more highly expressed by the metacercariae and NEJ 1 h stages compared with greater expression of the aquaglyceroporin-like genes (*aqp-3-like* and *aqp-9-like*) by the NEJ 3 h and 24 h. These data indicates that the regulation of water and solutes is particularly important for the early fluke lifecycle stages that are reliant on oxygen diffusion across the tegument as part of their metabolism.

The role of aquaporins within the parasite tegument is further highlighted by immunohistochemical studies of related trematodes *Fasciola gigantica* and *S. mansoni* that have localized an aquaporin to the adult parasite tegument and within the epithelial lining of the testes and ovary (39, 40). Sequence comparison of the aquaporins from *F. gigantica* suggests that these function as classical aquaporins capable of water transport, albeit due to changes to the first NPA motif to TAA, their efficiency may be diminished (40). Our analysis of the *F. hepatica* aquaporins shows that one sequence is homologous to the *F. gigantica* AQP, with a modification of the first NPA motif to TAA, termed here FhAQP-1. Except for one protein, termed here FhAQP-2 that has the first NPA motif modified to NPS, the remaining *F. hepatica* AQPs contain the two classical NPA motifs found within transmembrane domains. Interestingly, characterization of the *S. mansoni* aquaporin, SmAQP, revealed that this is an aquaglyceroporin capable of transporting mannitol, fructose, alanine, and lactate, indicating potential roles in nutrient uptake and waste excretion, as well as osmotic regulation (39). Sequence analysis, specifically focusing on the aromatic/arginine selectivity filter and the Froger's residues indicated that *F. hepatica* has four aquaglyceroporins that show homology to SmAQP and may also transport similar solutes (supplemental Fig. S3).

##### Metacercariae are Metabolically Primed for Excystment and Tissue Invasion

The infectivity of *F. hepatica* is reliant on the metacercarial stage that must survive encysted on vegetation for an indefinite period before being ingested by the mammalian definitive host. During this time the parasite must contend with an array of environmental stresses. Our gene expression analysis has identified metacercariae-up-regulated genes associated with the GO term GO:0050896 response to stimulus, specifically GO:0006950 responses to oxidative stress/stress and GO:0009650 UV protection, consistent with the need for the metacercariae, despite being encased in a protective cyst, to shield themselves from ambient conditions (Fig. 2; supplemental Fig. S4).

**Fig. 3.**
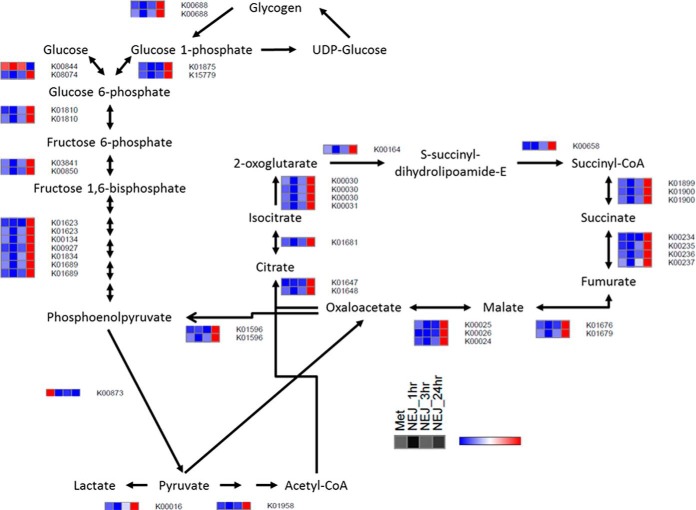
**Graphical representation of transcript expression for the TCA and glycolysis/gluconeogenesis KEGG pathways represented as heatmaps for the metacercariae and newly excysted juveniles (NEJ) 1 h, 3 h, and 24 h post-excystment.** Relative expression is shown by a blue to red scale depicting low to high levels of expression, respectively.

Metacercariae, like other free-living *F. hepatica* stages (eggs, miracidia), are nonfeeding such that they rely on endogenous energy stores in the form of glycogen and therefore, have a defined time in which they must infect a host before their reserves are depleted. Metacercariae obtain oxygen from their environment but as soon as the parasite enters the host tissue, rapid growth and development ensues and oxygen diffusion across and within the parasite becomes limited by parasite size. This results in a gradual switch from aerobic energy metabolism, particularly in the deeper tissues, via aerobic acetate production using the Tricarboxylic Acid Cycle (TCA) pathway, to anaerobic dismutation (41). We found that at both the transcript and protein level that components of the pathways involved in aerobic energy metabolism dominate in metacercariae and display comparable levels of transcription/expression to those of NEJ within 3 h post-excystment (Fig. 3; Supplemental Fig. S5). These data contradict the general view that the metacercarial stage of the parasite is biochemically dormant (41, 42) and could explain why the longevity of this stage in the field is dependent on ambient temperatures (5) and directly correlated with the age of the encysted parasites (43). Consistent with observations made half a century ago (6) our study suggests that the parasites are prepared to sense environmental cues posed by the changing salinity and increased CO_2_ tension of its new environment that will initially kick off the activation of the metacercariae. Possible sensing mediators are water/solute transporters, particularly aquaporins, that are expressed within their surface tegument.

Once ingested, the metacercariae need to trigger a mechanism to ensure they can quickly escape from the cyst. The outer cyst wall is removed by the contents of the host stomach, particularly acid proteases, which activates the parasites from their resting phase within the inner cyst. In the duodenum, the parasites quickly emerge from the inner cyst wall by releasing their caecal contents that are loaded with cathepsin cysteine proteases. Analysis of the somatic proteome of the metacercariae identified five major cysteine proteases representing the cathepsin L3 clade and the cathepsin B proteases, FhCB1, FhCB2, FhCB3, and FhCB9 (supplemental Table S6). Various groups including ours have reported the presence of cathepsin L3 and cathepsins B1, B2, and B3 in NEJ (44–46) but FhCB9 has only previously been identified at the transcript level. The identification of this protease within both the metacercariae somatic proteome and the NEJ secretome, implies that this novel protease plays an important function during the early stages of infection. The overall abundance of these cathepsin proteases within the somatic proteome of the nonactivated metacercariae indicates that the parasite has amassed the proteases it requires for rapid excystment on stimulation within the mammalian gut. Immunolocalisation studies using resin embedded sections have shown the presence of cysteine proteases within the metacercarial stages of *F. gigantica* (47, 48). Thus, the storage of these proteases may begin within the intermediate snail host as in the related trematode *Schistosoma* spp (49). and/or following metacercarial encystment on vegetation.

In addition to the cathepsin L and B proteases, three asparaginyl endopeptidases (legumains) were also discovered as storage proteins in metacercariae; based on protein abundance estimates, these proteases are present within the somatic proteome at twice the amount of the cathepsin L and B proteases (supplemental Table S6). Our mass spectrometry data included matches to peptides derived from the N-terminal prosegment domains of both cathepsin B and L proteases confirming that these enzymes are stored as inactive zymogens. Thus, the NEJ parasites employ legumains to “kick-start” the catalytic trans-activation of the zymogen forms of cathepsin L and B proteases (45, 50). This sets off an amplification process of trans- and auto-activation to quickly generate the mature active forms and thus the coexpression of all these proteases is consistent with a mechanism of a fast-acting and organized tissue-degrading process.

However, unexpectedly the most abundant of the asparaginyl endopeptidases, Fh_legumain-1, has the classical active site cysteine residue replaced by a serine residue. A similar substitution is found in a legumain of *S. mansoni* and is responsible for its lack of hydrolytic activity (51). Therefore, it is unlikely that Fh_legumain-1 plays a role in protease activation and its function remains an enigma; however, it could act as a regulator of activation by binding to cysteine proteinases and preventing their activation by other legumains or cysteine proteases.

Intriguingly, the metacercariae up-regulates two genes associated with the GO term GO:0032496 response to lipopolysaccharide (LPS), which could be a precaution against exposure to bacteria of the gut microbiome, particularly the outer membrane surface LPS, when the parasite emerges and battles to find the intestinal wall. These proteins may also be important for regulating or preventing the induction of the host's pro-inflammatory innate response to bacterial LPS that may be carried with the parasite as it burrows through the intestinal wall. Although *Fasciola* is only present within the mammalian gut for a short time relative to its lifecycle (∼2 h) there is no evidence from histological and immunological studies for the induction of pro-inflammatory responses, such as the recruitment of neutrophils and macrophages, into the intestinal wall (52). The expression of genes that respond to LPS could imply that *F. hepatica* can influence the make-up of the host gut microbiota, as shown in studies of the interactions of gut-dwelling nematodes with the host intestinal microbiome and intestinal mucins (53–56).

##### Major Metabolic Changes are Observed in Both Transcriptome and Somatic Proteome of the NEJ

**Fig. 4.**
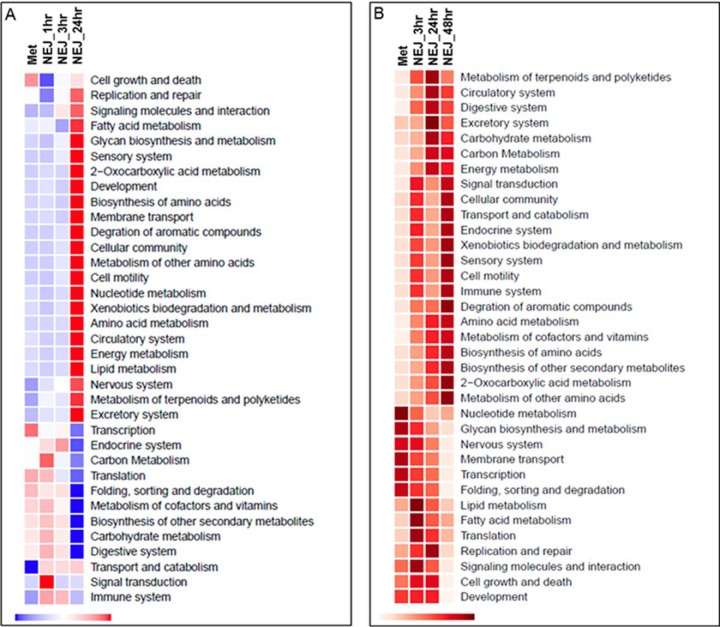
***F*. hepatica metabolism.** *A*, Graphical representation of the transcription of genes associated with the metabolic KEGG modules (ko00001) across the *F. hepatica* lifecycle within the first 24 h, normalizing the global patterns of expression at the KEGG module level. Relative expression is shown by a blue to red scale depicting low to high levels of expression, respectively. *B*, Graphical representation of the somatic protein abundance corresponding to the proteins associated with the metabolic KEGG modules (ko00001) within the infective stage, metacercariae and the NEJ up to 48 h post-excystment. The global patterns of expression were normalized at the KEGG module level. Relative protein abundance is shown by light to dark red scale, depicting low to high protein abundance, respectively.

Analyses of the pathways related to aerobic metabolism revealed that metacercariae have comparable levels of gene transcription to the NEJ 1 h and 3 h, which are motile and prepared for tissue invasion. Quantitative metabolic fingerprint (QMF) analysis, whereby reads mapped to genes annotated by KEGG were grouped together per KEGG module/pathway and normalized to the total KEGG annotated reads, revealed that across all pathways, the metacercariae had comparable levels of gene transcription to the NEJ up to 3 h post-excystment (Fig. 4*A*). A similar pattern was also observed at the protein level within the somatic proteome (Fig. 4*B*). Several pathway modules were found to be up-regulated during the metacercariae and NEJ 1 h and 3 h. For example, consistent with pathways relating to aerobic metabolism, the module carbohydrate metabolism, that includes the glycolysis/gluconeogenesis pathway (map00010) and the TCA cycle (map00020), was up-regulated compared with the NEJ 24 h. Similarly, the module relating to protein folding, sorting and degradation, that also encompasses protein export (map03060), protein processing in the endoplasmic reticulum (map04141) and SNARE interactions in vesicular transport (map04130), was found to be more highly expressed at both the transcript and protein level, for the metacercariae and the NEJ 1 h and 3 h. Interestingly, components of the transcription module that encompasses RNA polymerase (map03020) and basal transcription factors (map03022) was observed at higher levels within the metacercariae for both gene transcript and protein, supporting our the premise that the metacercariae are transcriptionally active. These data are consistent with a parasite producing proteins to aid its invasion and migration through the mammalian host.

As evidenced by the network analysis (Fig. 1), the NEJ 24 h display increased gene transcription compared with the metacercariae and NEJ 1 h and 3 h. A large component of these genes is related to an increase in transcription of metabolic genes, relating to a variety of KEGG pathway modules, including development, biosynthesis of amino acids, nucleotide metabolism, energy metabolism and the nervous system. This increased transcription correlated with the level of protein expression of these modules within the somatic proteome. Within 24 h, proteins involved in pathway modules associated with development and growth were observed, including DNA replication/repair and cell growth/death. By 48 h post-excystment, increased levels of proteins associated with amino acid metabolism and biosynthesis, the sensory system and xenobiotic biodegradation and metabolism were observed, suggestive of a parasite growing and sensing its environment.

To facilitate the increase in metabolism associated with infection, *F. hepatica* parasites must expend a high level of energy generated via the large glycogen stores present within parenchymal cells (57, 58). These stores are estimated to be depleted within 12 h of infection and must be replenished (58). Accordingly, key enzymes involved in glycogen metabolism and biosynthesis within the glycolysis/gluconeogenesis pathway are tightly regulated. Gene transcription of the glycolysis/gluconeogenesis pathway is up-regulated in the metacercariae and NEJ 1 h and 3 h. In comparison, analysis of the proteins/enzymes associated with these pathways revealed increased expression after 24 h (Fig. 4). Analysis of the specific enzymes within this pathway show that phosphofructokinase (related to glycogen breakdown) and fructose 1,6, bisphosphatase (related to glycogen synthesis) are switched on in high abundance within 24 h post-excystment. The levels of fructose 1,6, bisphosphatase decreased after 24 h indicating that at 48 h the NEJ are metabolizing the glycogen they have synthesized (supplemental Table S6). These specific enzymes have been shown to be important for glycogen metabolism in the dormant *C. elegans* dauer stage, which display high phosphofructokinase activity, indicating high levels of glycogen metabolism, and suppressed levels of fructose 1,6, bisphosphatase suggesting the suppression of glycogen resynthesis (33, 59).

##### Gut Development and Neoblast Proliferation Indicates Rapid Growth and Development in the First 2 days of Infection

**Fig. 5.**
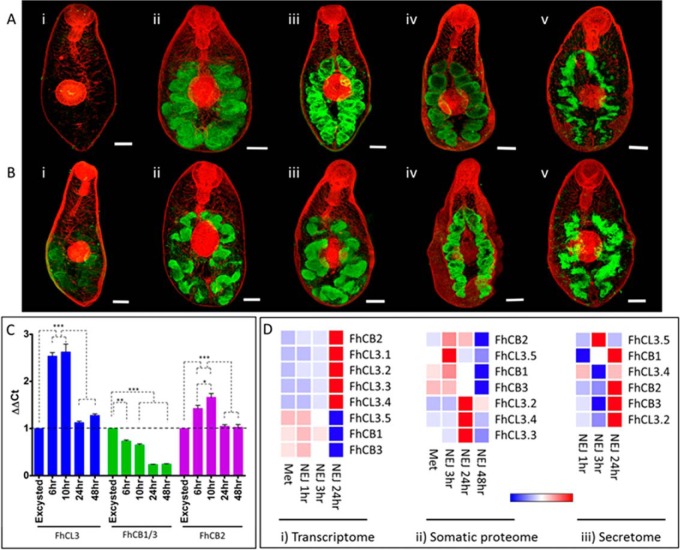
**Analysis of NEJ-specific *F. hepatica* cathepsin L (FhCL3) and B (FhCLB) cysteine proteases.** *A*, Immunolocalization of FhCL3 in NEJ by CSLM, over a time-course of 48 h, represented by green fluorescence (FITC staining) within the NEJ gut. *B*, Immunolocalization of FhCB in NEJ by CSLM, over a time-course of 48 h, represented by green fluorescence within the NEJ gut (FITC staining). Time-course: (i) at excystment, (ii) 1 h post-excystment, (iii) 6 h post-excystment, (iv) 10 h post-excystment, (v) 24 h post-excystment. All specimens were counter-stained with phalloidin- TRITC to stain muscle tissue (red fluorescence) and provide structure. Scale bars = 20 μm. *C*, Relative fold expression of cathepsin L (FhCL3) and B (FhCB1/CB3 and FhCB2) genes over a time-course of 48 h normalized to expression at NEJ excystment relative to a GAPDH reference, with S.E. Statistical analysis was carried out using One Way ANOVA with Tukey's post hoc test (*p* < 0.05: *; *p* < 0.01: **; *p* < 0.001: ***). *D*, Graphical representation of gene transcription (i), protein abundance within the somatic proteome (ii) and protein abundance within the secretome (iii). Relative expression/abundance is shown by a blue to red scale, depicting low to high levels of expression/abundance, respectively.

Access to readily available nutrient is believed to be a pivotal selection pressure in driving the evolution of parasitism and thus the rapid development of a functional digestive tract capable of degrading host macromolecules is viewed as essential for parasites to adapt to their host (60). In many trematode parasites, proteolytic enzymes produced by the gastrodermal cells are employed for invasion and feeding on host tissues and thus can be used as markers of gut development. We have previously shown that *F. hepatica* is unique, compared with other helminths, in that it relies exclusively on cathepsin L and cathepsin B cysteine proteases to perform these duties. Indeed, genes encoding these proteases have expanded by gene duplication and then functionally diverged to form large families with overlapping and novel specificities (9, 61, 62). Here we have used specific antibodies to probe the *F. hepatica* NEJ parasites over a time-course of 48 h in culture for the presence of two major cathepsins identified by our transcriptomic and proteomic analysis, namely FhCL3 and FhCB (FhCB1, FhCB2, FhCB3). Both proteases are highly expressed within the bifurcated parasite gut. FhCB was localized within the NEJ parasites immediately after they emerged from their cyst (Fig. 5), indicating that these proteases are stored within the metacercariae in preparation for the excystment process. Excystment requires reducing conditions that likely activate the FhCB proteases, and studies have shown that inhibitors of cysteine proteases prevent excystment (45). The pattern and intensity of protein expression localized within the whole-mount NEJ correlate with the gene transcript data showing greater transcription of FhCB within the newly excysted parasites compared with NEJ at 6 h, 10 h, 24 h, and 48 h post-excystment (Fig. 5).

By contrast, the expression of FhCL3 within the gut appears to be delayed compared with FhCB as expression of this protease is up-regulated about 1 h after excystment. This finding suggests that FhCL3 is not required for excystment but is secreted at a time when the parasite invades the intestinal wall. Consistent with this idea is our previous reports highlighting the unique collagenolytic-like activity of FhCL3 whereby modifications within the active site allows the protease to efficiently cleave within the left-handed coils specific to collagen (63–65). Furthermore, qPCR data show that, the greatest transcription of FhCL3 is at 6 and 10 h post-excystment when compared with transcription at excystment (Fig. 5*C*), in agreement with our suggestion that these proteases are used in tissue migration. The digestion of collagen by FhCL3, in combination with the proteolytic activities of FhCB, would provide the parasite with a very effective digestive mechanism for tissue breakdown and digestion.

The importance of the cysteine proteases in host invasion is also revealed by our proteomic analysis of the NEJ secretomes which found that FhCB (FhCB1, FhCB2, and FhCB3) and FhCL3 proteases comprise the major components of the NEJ secreted proteins, representing ∼20% of the total protein secreted in the first 24 h post-excystment (supplemental Table S7; see below). Their presence within the bifurcated gut of the parasite suggests that the gastrodermal cells are critical not only for the digestion and absorption of nutrients for the parasite but also in providing the effectors required for excystment and digestion of interstitial proteins such as collagen, laminin and fibronectin (64, 66). The effective tunneling activity of the NEJ was described in the microscopical studies performed by Dawes and Hughes (67) over 50 years ago and demonstrated that the parasite's physical activity and secretory machinery degrade not only interstitial matrices of the intestinal wall but also cellular tissue, including muscle. With the benefit of “omics” technologies, we now have a greater understanding of these events at a molecular level.

**Fig. 6.**
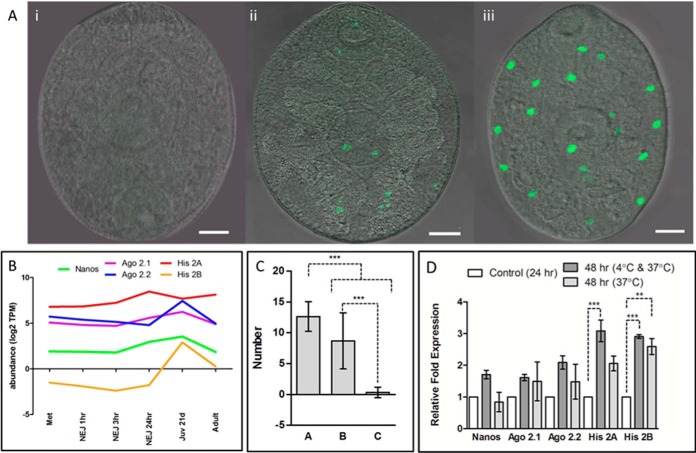
**Proliferation of neoblast-like cells during the first 48 h post excystment.** *A*, Incorporation of 5-ethynyl-2-deoxyuridine (EdU) by the proliferative neoblast-like cells highlighted by green fluorescence; (i) NEJ following 24 h culture with Edu at 4 °C, (ii) NEJ following 24 h culture at 4 °C followed by 24 h culture with Edu at 37 °C, (iii) NEJ following 48 h culture with Edu at 37 °C. Scale bars = 20 μm. *B*, Graphical representation of the expression of genes associated with neoblast-like cells in transcripts per million (TPM) across the *F. hepatica* lifecycle, displayed on a log2 scale. *C*, Number of neoblast cells identified after (a) NEJ incubated for 48 h with 5-ethynyl-2-deoxyuridine (EdU) at 37 °C, (b) NEJ cultured for 48 h at 37 °C, with the addition of Edu for the last 24 h of culture, (c) NEJ cultured for 48 h, the first 24 h at 4 °C, following by the remaining 24 h culture at 37 °C with the addition of Edu. The differences between these groups were statistically significant (*p* < 0.001: ***). *D*, Relative fold expression of genes associated with neoblast-like cells normalized to expression at 24 h relative to a GAPDH reference, performed in duplicate, with S.E. Statistical analysis was carried out using One Way ANOVA with Tukey's post hoc test (*p* < 0.01: **; *p* < 0.001: ***).

Another indicator of development and growth of NEJ within the first 24 to 48 h post-excystment is the rapid proliferation of neoblasts (pluripotent stem cells), also observed in the recent study by McCusker *et al.* (68). Transcriptome analysis revealed that genes associated with neoblasts such as *nanos*, *argonaute 2* (*Ago 2.1* and *Ago 2.2*) and *histone 2A* (*His 2A*) (29) are constitutively expressed in parasites from NEJ to adult stages within the mammalian host but have increased transcription in the juvenile parasites. The exception is histone 2B, (*His 2B*) that is only “switched on” at 21 days post infection, based on gene transcription levels of less than 2 TPM prior to this stage (Fig. 6; supplemental Fig. S6; supplemental Table S2). The dramatic up-regulation of these genes begins 24 h post-excystment, suggesting that the parasites are preparing for the invasive migration into the nutrient-rich liver after which the parasite undergoes tremendous growth and development. The increased transcription levels are mirrored by the expansion of neoblasts observed in NEJ (Fig. 6); the number of neoblasts observed increased with the age of the parasite from <2 discernable neoblasts in NEJ 24 h to an average of 12 neoblasts in NEJ 48 h.

Our studies suggest that neoblast proliferation may also be correlated with temperature increases, as we found that the number of neoblasts observed per NEJ was significantly reduced (∼2/NEJ) when NEJ were first incubated for 24 h at 4 °C followed by 24 h at 37 °C compared with 48 h culture at 37 °C (∼12/NEJ) (*p* < 0.001; Fig. 6). However, despite the smaller number of neoblasts within these NEJ, qPCR analysis showed that *histone 2A* transcription was significantly up-regulated (*p* < 0.05) in the cold-induced NEJ compared with those cultured at 37 °C. Studies in plants have shown an epigenetic role of histones (H2A and H2B) in relation to temperature; incorporation of the variant H2AZ into nucleosomes is favored in cooler temperatures, which is important for the correct detection of ambient temperature (69) and H2B de-ubiquitination has been shown to be involved in regulating flowering (70). The lack of transcription of the other neoblast-like genes may imply that histones 2A/2B are playing a role in regulating their proliferation, specifically down-regulating their expression until a more biologically appropriate temperature is reached.

##### The NEJ Secretome is Dominated by Just 10 Proteins

**Fig. 7.**
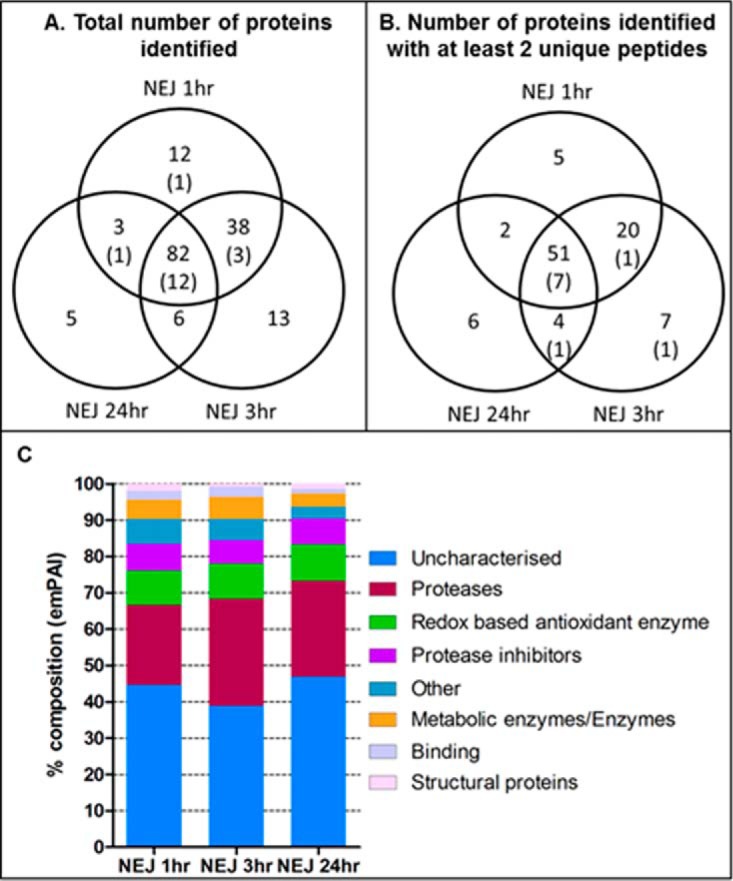
**Proteins identified by NEJ secretome analysis.** *A*, Venn diagram representing the mean value of proteins identified within biological replicates of NEJ secretomes (1 h, 3 h, and 24 h post-excystment; 4, 3, and 3 biological replicates, respectively). *B*, Venn diagram representing those proteins across all three NEJ secretomes with a cutoff of at least 2 unique peptides (biological replicates as above). Those proteins that were uncharacterised are included in brackets. *C*, Graphical representation of the composition of the NEJ secretomes, based on the emPAI abundance of the different proteins types as a proportion of the total protein secreted.

The ability of the *F. hepatica* parasites to successfully invade tissues of their mammalian hosts, while evading the host's defenses, is likely to be primarily mediated through the proteins they excrete/secrete (ES proteins). Following the development of new protocols that facilitated the recovery of proteins from parasite excystment media, we report the first proteomic analysis of ES proteins collected at 1 h and 3 h post-excystment (Fig. 7) and compare these to proteins released after a subsequent 24 h culture. A total of 159 proteins were identified, compiled from 135, 139 and 96 proteins of the NEJ 1 h, 3 h and 24 h postexcystment secretome samples, respectively (Fig. 7). Based on acceptance criteria of two unique peptide matches, a 95-protein subset was used for further analysis (Fig. 7; supplemental Table S7). This 95-protein subset included a range of proteases, protease inhibitors, redox-based antioxidant enzymes, metabolic enzymes, structural proteins and proteins involved in binding (Fig. 7), a profile consistent with earlier studies reported by us (45) and others (71). Interestingly, the range of proteins identified in the NEJ secretome is far more diverse than that described for the adult fluke secretome (45, 72, 73), which likely reflects their distinct interactions with their host. Although the migratory NEJ stage encounters a diverse range of molecules, including bacterial cells within the gut and immune cells within the peritoneal compartment and liver, the adult parasites reside in the bile ducts safe from the host's immune response (5), where the bile acids can depress both the cell-mediated and humoral immune responses (74). This probably allows the adult parasites to redirect the energy not expended in the fight against immune attack, toward the production of massive quantities of eggs per day (∼24,000/fluke per day; (75).

**Fig. 8.**
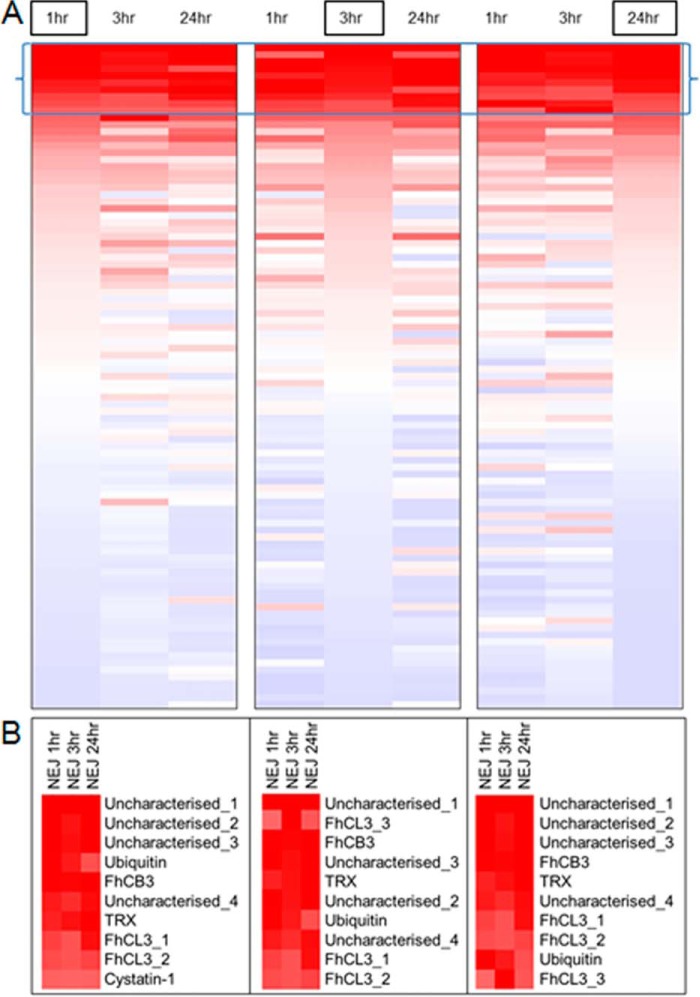
**Comparison of protein abundance for the NEJ 1 h, 3 h, and 24 h secretomes.** *A*, Each NEJ secretome (1 h, 3 h, and 24 h) is represented as a heatmap ranked by emPAI score for each sample separately, indicated by the boxed sample name. Up-regulation: red; Down-regulation: blue. The top 10 proteins in terms of abundance are indicated within the brackets. *B*, Highlighted section depicting the top 10 proteins from each heatmap including protein annotation.

Deeper analysis of protein abundance showed that although each NEJ secretome sample shared a similar cohort of proteins the expression profile differed among the different time-points (Fig. 8). Most notably, 10 proteins were found to be particularly abundant and represented ∼70% of the total secretome for each time point. This group consisted of the cathepsins B and L proteases that localize to the gut within the NEJ (FhCB3 and FhCL3; Fig. 5; see above), a cysteine protease inhibitor, cystatin-1, the anti-oxidant, thioredoxin, as well as four uncharacterized proteins. Three of these uncharacterized proteins share structural resemblance to the cobalamin (vitamin B12) Intrinsic factor (Uncharacterized_2, Uncharacterized_3, and Uncharacterized_4; I-TASSER, (76). Correlating with their high abundance in the secretome, the genes encoding these 10 proteins also show high levels of transcription, and are present within the top 15% of genes transcribed by the metacercariae and NEJ stages (supplemental Fig. S6; supplemental Table S4). Importantly, transcripts encoding FhCL3, FhCB3 and the inactive Fh_legumain-1 were observed within the most highly expressed 1.6% of the NEJ transcriptomes. Thus, our collective transcriptome and proteome data emphasize the importance of this select group of highly secreted proteins for the invasion and establishment of *F. hepatica* infection.

Our extensive analysis of the somatic proteome for the metacercariae and NEJ identified a total of 1671 proteins. As discussed above, a large proportion of these proteins function as metabolic proteins involved in a variety of KEGG pathways (Fig. 3 and 4). Comparative analysis of our somatic proteome with the ES proteins revealed that 88 members of the NEJ ES protein profile were present within the somatic data. The seven ES proteins not found in the somatic proteomic data represent additional cathepsin B and legumain proteases, ubiquitin, a kunitz-type inhibitor, actin and an uncharacterized protein.

The study by Di Maggio *et al.* (71) analyzed the somatic proteome and secretome of NEJ 48 h of a North American isolate by gel-free mass spectrometry, resulting in the identification of 575 and 90 proteins, respectively. Comparative analysis between the Di Maggio study and the proteins identified as part of this study has shown that 513 somatic proteins overlap among the studies. By contrast, only a third of the ES proteins were shared, despite a similar number of secreted proteins identified in each study. The main differences pertain to the lack of anti-oxidant related proteins, such as fatty acid binding proteins, glutathione S transferases and superoxide dismutase and many serpins found by Di Maggio and colleagues. Conversely, their study identified a greater number of cathepsin L and legumain proteases, including cathepsin L4 (FhCL4), which was previously thought to play an intracellular role rather than being secreted (44, 45). The use of different isolates, number of NEJ parasites and culturing conditions, as well as the sensitivity of proteomic methods employed may account for the discrepancies among these studies.

##### Immunomodulatory Molecules

The secretion of molecules that modulate, manipulate and evade the activity of the mammalian host immune system is a critical means by which NEJ of *F. hepatica* prevent their elimination at a stage when they are most vulnerable. The myriad of the NEJ secreted molecules likely combine to confuse and/or modulate the immune responses of the host by targeting a variety of host innate response mechanisms and cells. Studies in murine models of infection have shown that parasite-mediated modulation of innate immune cell function, such as macrophages, is effected as quickly as 6 h following oral infection (S. Donnelly, personal communication). Several immunomodulatory molecules that have been isolated from adult parasites were detected within the NEJ secretomes and suggest that early stage parasites use a similar mechanism to evade the host immune response. Additionally, the abundant cathepsin L and cathepsin B proteases have also been shown to block the induction of Th1 responses, possibly by cleaving TLR 3 within endosomes of innate cells and by the prevention of the MyD88-independent, TRIF-dependent signaling pathways (77).

As the parasite invades and migrates through the mammalian host, it encounters a variety of different aerobic/anaerobic micro-environments, especially those generated by immune cells. To ensure its continued survival, the parasite has developed an anti-oxidant system that protects against the reactive oxygen and nitrogen species generated by both endogenous cellular metabolism and host immune cells (78). Key components of this redox based anti-oxidant system, specifically peroxiredoxin (FhPRX) and the related protein, thioredoxin (FhTRX) were among the most abundant proteins within the NEJ secretomes. Through the induction and recruitment of M2 alternatively activated macrophages, FhPRX is known to skew the host immune response toward a Th2 type response which is not protective against *F. hepatica* parasites (7, 8). FhTRX is the most abundant protein present within the NEJ 24 h and 48 h somatic proteomes, and is also within the top 5 proteins secreted by the NEJ 24 h. In contrast, the protein it reduces, FhPRX, was found to be marginally less abundant, found within the top 50 somatic proteins and top 15 secreted proteins (supplemental Table S6; supplemental Table S7). Intriguingly, the FhTRX-reducing enzyme, thioredoxin glutathione reductase (TGR), was found within the somatic proteome but not the secreted proteins. These data indicate that FhTRX is either reduced prior to its secretion or another component within the external environment is capable of reducing FhTRX to facilitate this process.

Piedrafita and colleagues (79) have previously reported that *F. hepatica* NEJ parasites are resistant to superoxide-mediated killing through the elevated levels of *F. hepatica*-specific superoxide dismutase (SOD) activity compared with *F. gigantica* NEJ. We identified FhSOD within both the NEJ somatic proteome and secretome, though at lower levels than FhTRX or FhPRX. Focusing specifically on the somatic proteome, FhPRX was ∼2-fold more abundant than FhSOD across the NEJ timepoints (3 h, 24 h, and 48 h), with FhTRX 9-fold more abundant than FhSOD at 3 h and 104-fold more abundant at 24 h and 48 h (supplemental Table S8). As this analysis is based on protein concentration rather than protein activity, it is possible that FhSOD is more active in smaller concentrations than FhTRX and FhPRX, though FhTRX and FhPRX activity was not tested as part of the Piedrafita *et al.* (79) study as a comparison. Nonetheless, we have shown that NEJ have a complete anti-oxidant armory to neutralize reactive oxygen species (ROS) released by the innate cell response early in infection and the importance of this system is indicated by the fact that these are major components of their secretome.

Another molecule of significance found in NEJ secretome is the 8 kDa helminth defense molecule (FhHDM). This protein shares structural similarity to mammalian anti-microbial or cathelicidin-like molecules, such as Cap18/LL-37, and is suggested to be a parasite mimic of these host immune regulators effecting both the innate and adaptive immune response (80–82). Transcriptomic analysis reveals that FhHDM is not only transcribed by the early NEJ but is particularly elevated at 24 h. Similarly, it was also detected in the secretome of the NEJ 24 h. We have previously shown that FhHDM binds directly to bacterial LPS, reducing its interaction with both LPS-binding protein (LBP) and the cell surface; this protects mice against LPS-induced inflammation by significantly reducing the release of inflammatory mediators from macrophages (80). Thus, FhHDM may serve to protect the host from excessive inflammation that would otherwise be induced by translocation of bacteria and their toxins during penetration of the intestinal wall by the NEJ.

Although *F. hepatica* parasites are known to inhibit the classical and alternative complement pathways (83, 84), the mechanism by which they do so is unknown. Transcriptome analysis of host liver and peripheral blood mononuclear cells (PBMC) has shown that the genes associated with the complement cascade system are upregulated during *F. hepatica* infection (85, 86), which may be in response to the parasite surface or secreted proteins that can inhibit this process. In this regard, we have detected paramyosin, a muscle protein found on the surface of helminth parasites, within the NEJ secretomes and in the top ten most abundant proteins within the NEJ somatic proteomes. In the related trematodes *S. mansoni* (87, 88) and *Clonorchis sinensis* (89) and in the nematode *Trichinella spiralis* (90), paramyosin has been shown to bind several components of the complement cascade, such as C1q and C8 and C9, and thereby inhibit both the classical and alternative complement pathways. The inhibition of the complement cascade by *F. hepatica* could also be mediated through CD59-like proteins exposed on the parasite tegumental surface (91) and secreted within the ES protein fraction that prevent the formation of the complement membrane attack complex (MAC), another possible host mimicry mechanism. Shi and colleagues (92) have shown that nine CD59-like genes are present within the *F. hepatica* genome; seven corresponding to the FhCD59–1 group found within the tegumental proteome (91) and a single gene corresponding to FhCD59–2 and FhCD59–3, respectively. Consistent with qPCR analysis of these genes by Shi *et al.* (92), our analysis shows that FhCD59–2, which is homologous to the surface-associated CD59-like protein from *S. mansoni*, shows higher gene transcription within the NEJ stage.

More recently, Japa and colleagues (93) reported a *Fasciola* immunomodulatory molecule that is a member of the activin/transforming growth factor-like (TGF-b) family (termed FhTLM). They showed that this parasite-derived cytokine was abundantly transcribed by the NEJ based on qPCR data. It is noteworthy that we observed very low levels of transcription of this gene and did not detect FhTLM protein within the secreted proteins. Further investigation is therefore required to interpret these differences and the putative role of the FhTLM molecule in the immunoevasion strategies of the parasite.

## CONCLUSIONS

The recently sequenced draft genomes of *F. hepatica* (9, 10) have paved the way for investigative studies discovering new targets for novel drug or vaccine strategies. They have also revealed that this parasite is highly polymorphic (9), which is consistent with recent analyses of UK *F. hepatica* isolates that found high levels of genetic diversity in the field (94). Our goal here was to obtain a more dynamic picture of the parasite's biology, growth and development at time-points corresponding to early stages of infection by supporting the genomic information with transcriptome and proteome data. This integrated data set will allow future investigations into how polymorphisms and genetic differences among parasites influence the infection success rate of these parasites, their adaptability to different mammalian hosts as well as their potential to develop resistance to new control measures.

Our analysis of the *F. hepatica* NEJ transcriptomes/proteomes provides a comprehensive and dynamic view of infection at these early stages of the parasite life cycle, and provides a firm foundation to begin the process of targeting the pathways and molecules described in the search for new anti-*Fasciola* treatments that can prevent serious damage associated with acute disease. Similar analysis of other *F. hepatica* isolates is warranted as it could reveal the molecular basis behind our observed variations in fitness to infect and develop within the variety of mammalian hosts. Interestingly, the recent genome sequencing of American isolates of *F. hepatica* has also revealed the presence of *Neorickettsia* endobacteria (10). The similarity between the *Neorickettsia* genomes found within *F. hepatica* isolates of North and South America with respect to the genetic heterogeneity of the fluke isolates implies that this endobacteria may have been acquired recently. Several species of *Neorickettsia* carried by parasites are considered the causative agents of disease (95). Therefore *F. hepatica* could potentially be a carrier for a pathogenic endobacteria that could be transmitted from fluke to host, and warrants further investigation.

## DATA AVAILABILITY

The transcriptome data sets supporting the conclusions of this article are available in the European Nucleotide Archive repository, PRJEB6904; http://www.ebi.ac.uk/ena/data/view/PRJEB6904. The mass spectrometry proteomics data have been deposited to the ProteomeXchange Consortium via the PRIDE (103) partner repository with the data set identifier PXD007255 and 10.6019/PXD007255.

## Supplementary Material

Supplemental Data

## Footnotes

^1^ The abbreviations used are:

NEJ: Newly excysted juvenile
CSLM: confocal scanning laser microscopy
EdU: 5-ethynyl-2-deoxyuridine
emPAI: Exponentially Modified Protein Abundance Index
ES: excreted/secreted proteins
GAPDH: glyceraldehyde 3-phosphate dehydrogenase
GO: Gene Ontology
HDM: Helminth Defence Molecule
HSP: Heat Shock Protein
KEGG: Kyoto Encyclopedia of Genes and Genomes
LPS: Lipopolysaccharide
PBMC: Peripheral Blood Mononuclear Cells
PRX: Peroxiredoxin
ROS: Reactive Oxygen Species
RNASeq: RNA sequencing
SOD: superoxide dismutase
TCA: Tricarboxylic Acid Cycle
TPM: transcripts per million
TRX: Thioredoxin.
