# Supplementary material for: Infection by the Helminth Parasite Fasciola hepatica Requires Rapid Regulation of Metabolic, Virulence, and Invasive Factors to Adjust to Its Mammalian Host
Source: Mol Cell Proteomics. 2018 Jan 10;17(4):792–809. doi: 10.1074/mcp.RA117.000445 (PMC5880117; doi:10.1074/mcp.RA117.000445)
Supplement: Supplemental Data [file supp_17_4_792__index.html]

Infection by the helminth parasite Fasciola hepatica requires rapid regulation of metabolic, virulence, and invasive factors to adjust to its mammalian host — Running the gauntlet: Parasite infection of mammalian host — Infection by the Helminth Parasite Fasciola hepatica Requires Rapid Regulation of Metabolic, Virulence, and Invasive Factors to Adjust to Its Mammalian Host — Running the Gauntlet: Parasite Infection of Mammalian Host — Supplemental Data 

# Infection by the Helminth Parasite *Fasciola hepatica* Requires Rapid Regulation of Metabolic, Virulence, and Invasive Factors to Adjust to Its Mammalian Host

## Supplemental Data

- Supplemental Figures S1-S7 - Supplemental Figures S1-S7
- Supplemental Table S1 - Comparison between the F. hepatica databases used for Mascot searches
- Supplemental Table S2 - Analysis of neoblast-associated genes. Gene transcription represented as transcripts per million (TPM)
- Supplemental Table S3 - Comparison of the F. hepatica genome with other parasitic trematode genomes
- Supplemental Table S4 - Differential gene transcription between the F. hepatica metacercariae and NEJ 1hr, 3hr and 24hr post-excystment lifecycle stages, represented as transcripts per million (TPM).
- Supplemental Table S5 - Gene Ontology analysis of the gene clusters determined using the Biolayout network analysis software.
- Supplemental Table S6 - Identification of proteins within the somatic proteome of F. hepatica metacercariae and NEJ at 3hr, 24hr and 48hr post-excystment by LC-MS/MS.
- Supplemental Table S7 - Identification of proteins within the soluble secretome of F. hepatica NEJ at 1hr, 3hr and 24hr post-excystment by LC-MS/MS.
- Supplemental Table S8 - Differential expression of the F. hepatica antioxidants.
- Supplemental Material Legends - Legends to supplementary figures and tables
