# Supplementary material for: Infection by the Helminth Parasite Fasciola hepatica Requires Rapid Regulation of Metabolic, Virulence, and Invasive Factors to Adjust to Its Mammalian Host
Source: Mol Cell Proteomics. 2018 Jan 10;17(4):792–809. doi: 10.1074/mcp.RA117.000445 (PMC5880117; doi:10.1074/mcp.RA117.000445)
Supplement: Supplemental Data [file supp_RA117.000445_133638_1_supp_37265_p09v9y.pdf]

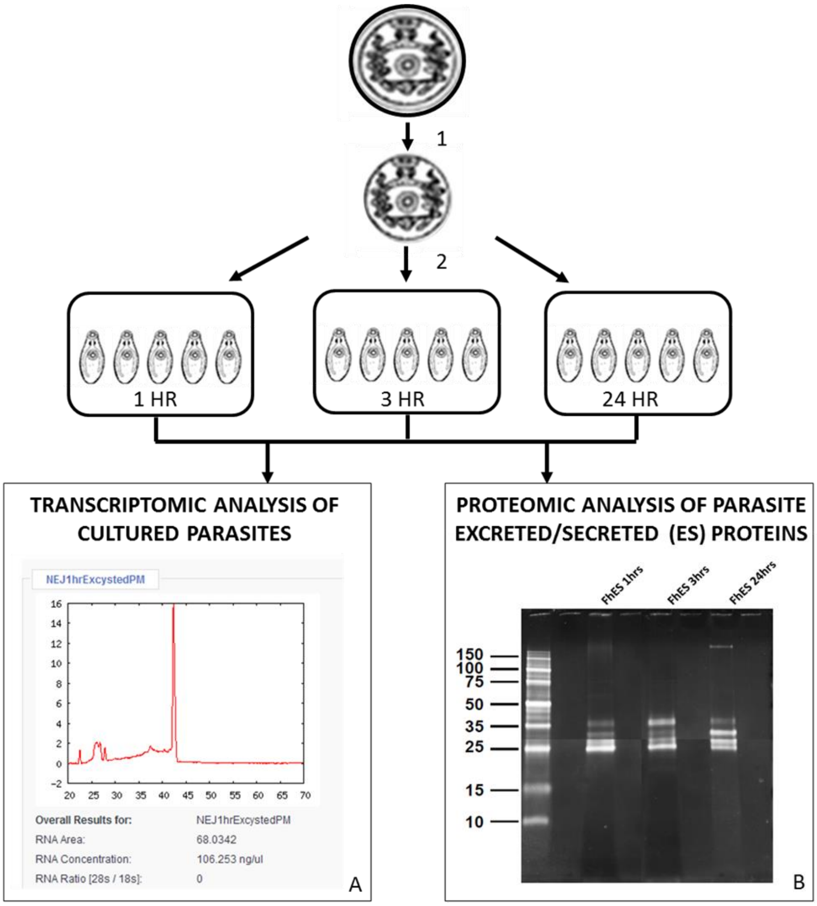

Supplemental Fig. S1: Graphical representation of the protocol used to recover the NEJ parasites and secreted proteins for transcriptomic and proteomic analysis. (1) The outer cyst wall was removed from the metacercariae. (2) Parasites were cultured in excystment media and recovered at 1hr, 3hr and 24hr post-excystment for RNA extraction. The excystment media and culture media were retained for proteomic analysis. (A) RNA integrity and concentration were confirmed using the Bioanalyzer 2100, with only one peak observed corresponding to the *F. hepatica* ribosomal RNA. (B) Secreted proteins isolated from the excystment media and culture media were concentrated and analysed by SDS PAGE electrophoresis (see Methods section).

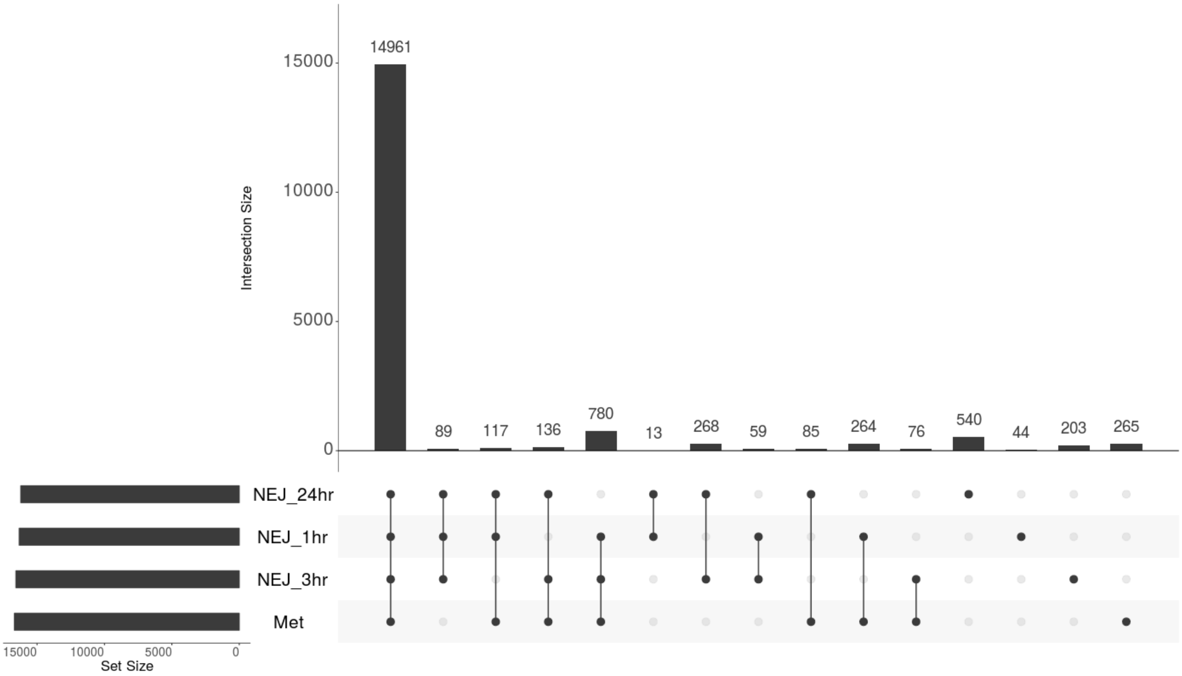

Supplemental Fig. S2: Upset plot depicting the number of genes identified by transcriptome analysis for the metacercariae, NEJ 1hr, 3hr and 24hr post-excystment lifecycle stages.

A

AQP	Identifier	P1	P2	P3	P4	P5
FhAQP-1	BN1106_s19269B000002	V	S	A	V	W
FhAQP-2	BN1106_s2080B000150	T	S	A	I	W
FhAQP-3	BN1106_s913B000263	G	S	A	I	W
FhAQP-4	BN1106_s913B000269	G	S	A	I	W
FhAQP-5	BN1106_s2498B000080	Y	D	R	F	W
FhAQP-6	BN1106_s2498B000081	Y	D	R	F	W
FhAQP-7	BN1106_s3330B000149	Y	D	R	F*	W*
FhAQP-8	BN1106_s12258B000030	Y	D	R	F	W

B

AQP	Identifier	1	2	3	4
FhAQP-1	BN1106_s19269B000002	Q	G	A	G
FhAQP-2	BN1106_s2080B000150	Q	G	A	G
FhAQP-3	BN1106_s913B000263	Q	G	A	G
FhAQP-4	BN1106_s913B000269	Q	G	A	G
FhAQP-5	BN1106_s2498B000080	E	G	A	G
FhAQP-6	BN1106_s2498B000081	Q	G	G	G
FhAQP-7	BN1106_s3330B000149	E	G	A	G
FhAQP-8	BN1106_s12258B000030	E	G	A	G

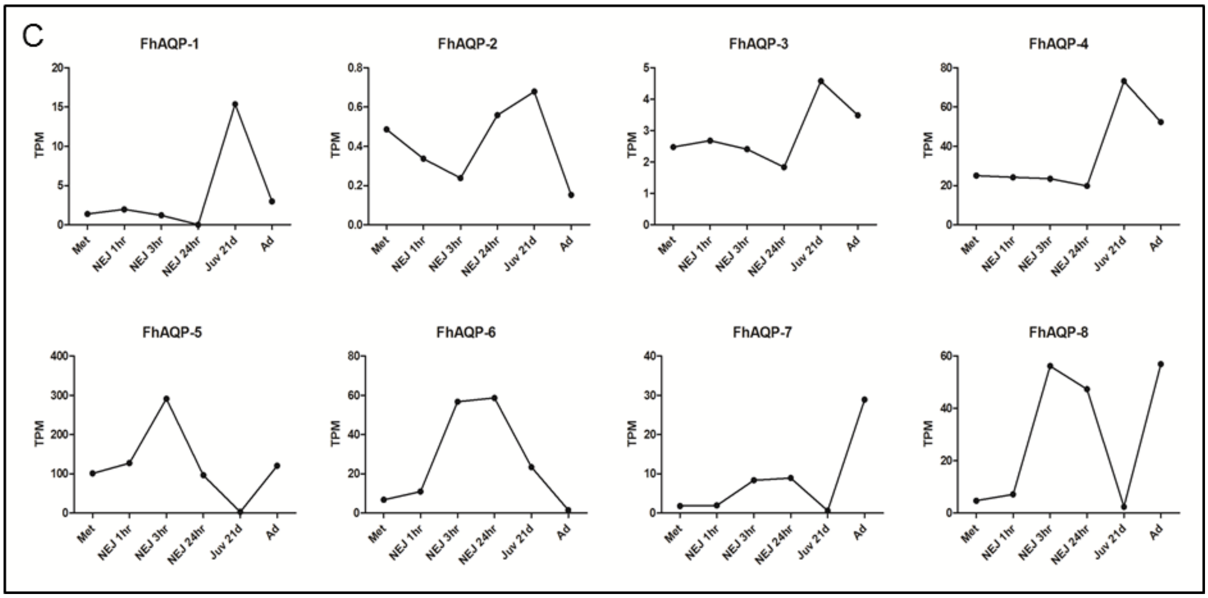

Supplemental Fig. S3: *Fasciola hepatica* transcribes a family of water channels (aquaporins). (A) Comparison of the Froger's residues across the *F. hepatica* aquaporins. (B) Comparison of the residues that comprise the aquaporin aromatic/arginine selectivity filter. (C) Graphical representation of gene transcription across the F. hepatica lifecycle represented as transcripts per million (TPM).

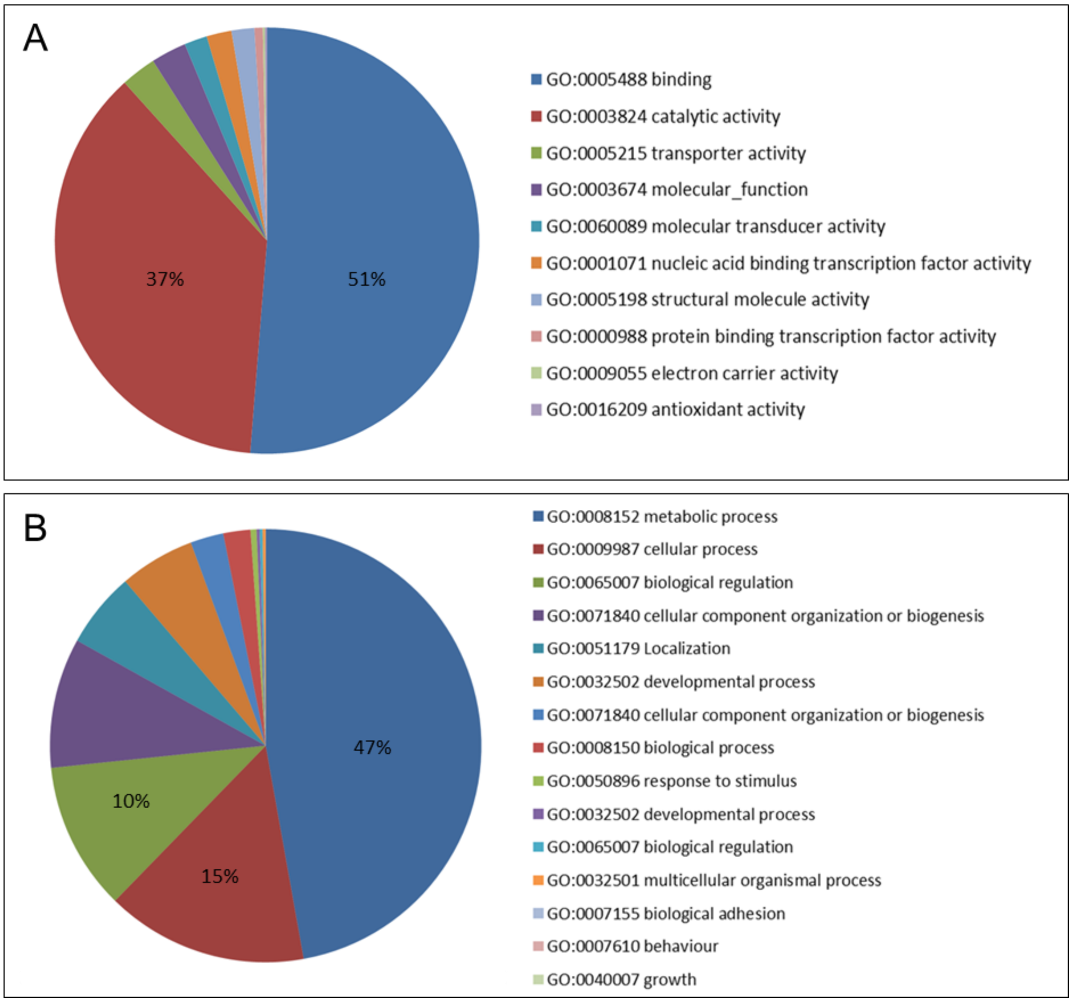

Supplemental Fig. S4. Graphical representation of Gene Ontology classification of genes expressed by *F. hepatica* metacercariae. (A) Genes grouped by Molecular Function classification. (B) Genes grouped by Biological Process classification. The proportion of genes represented by each GO term is shown (%).

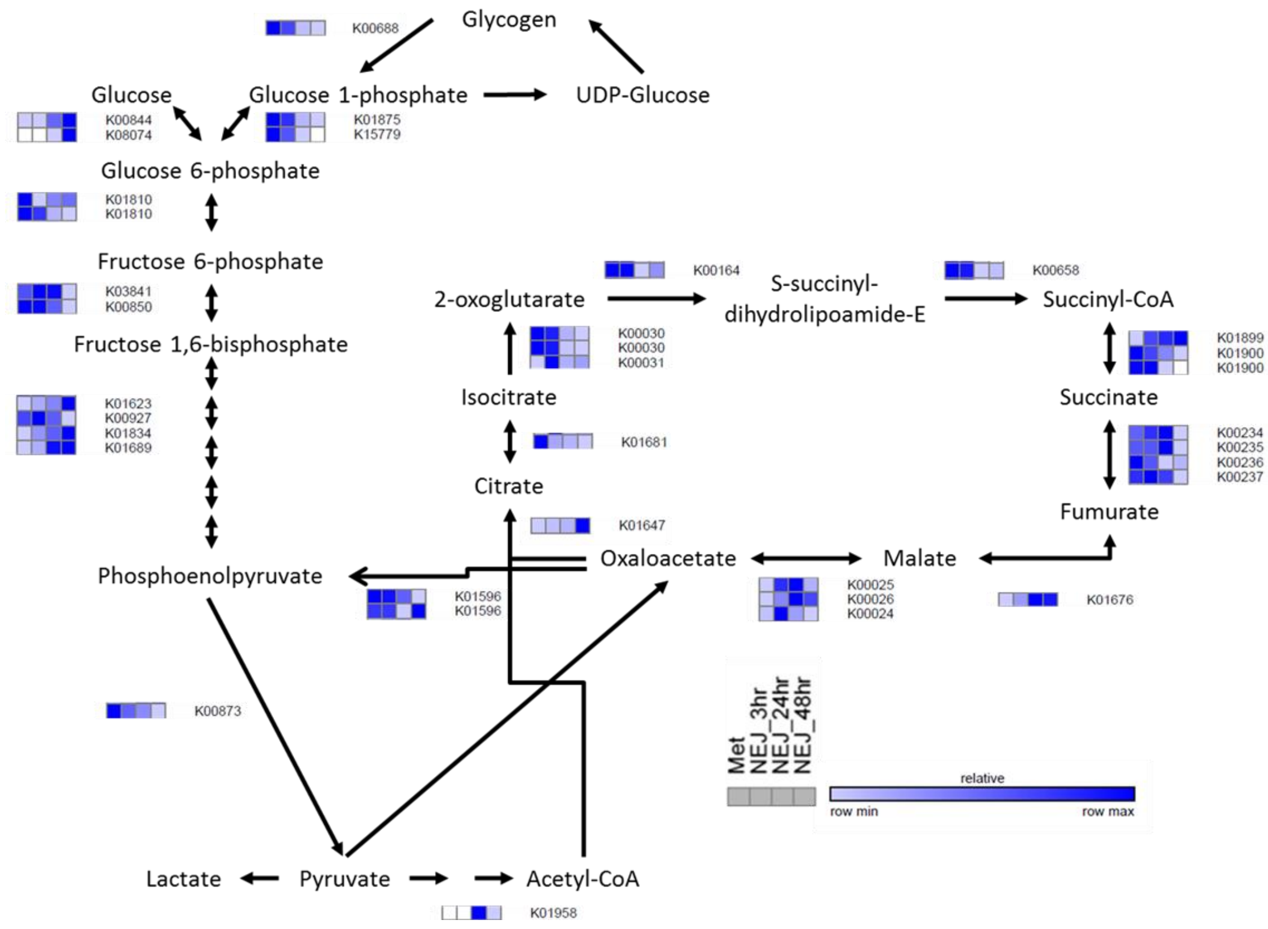

Supplemental Fig. S5: Graphical representation of somatic protein levels represented by emPAI value of the TCA and glycolysis/gluconeogenesis KEGG pathways represented as heatmaps for the metacercariae and newly excysted juveniles (NEJ) 3 hr, 24 hr and 48 hr post-excystment. Relative expression is shown on a blue scale, from light blue to dark blue, depicting low to high levels of protein expression, respectively.

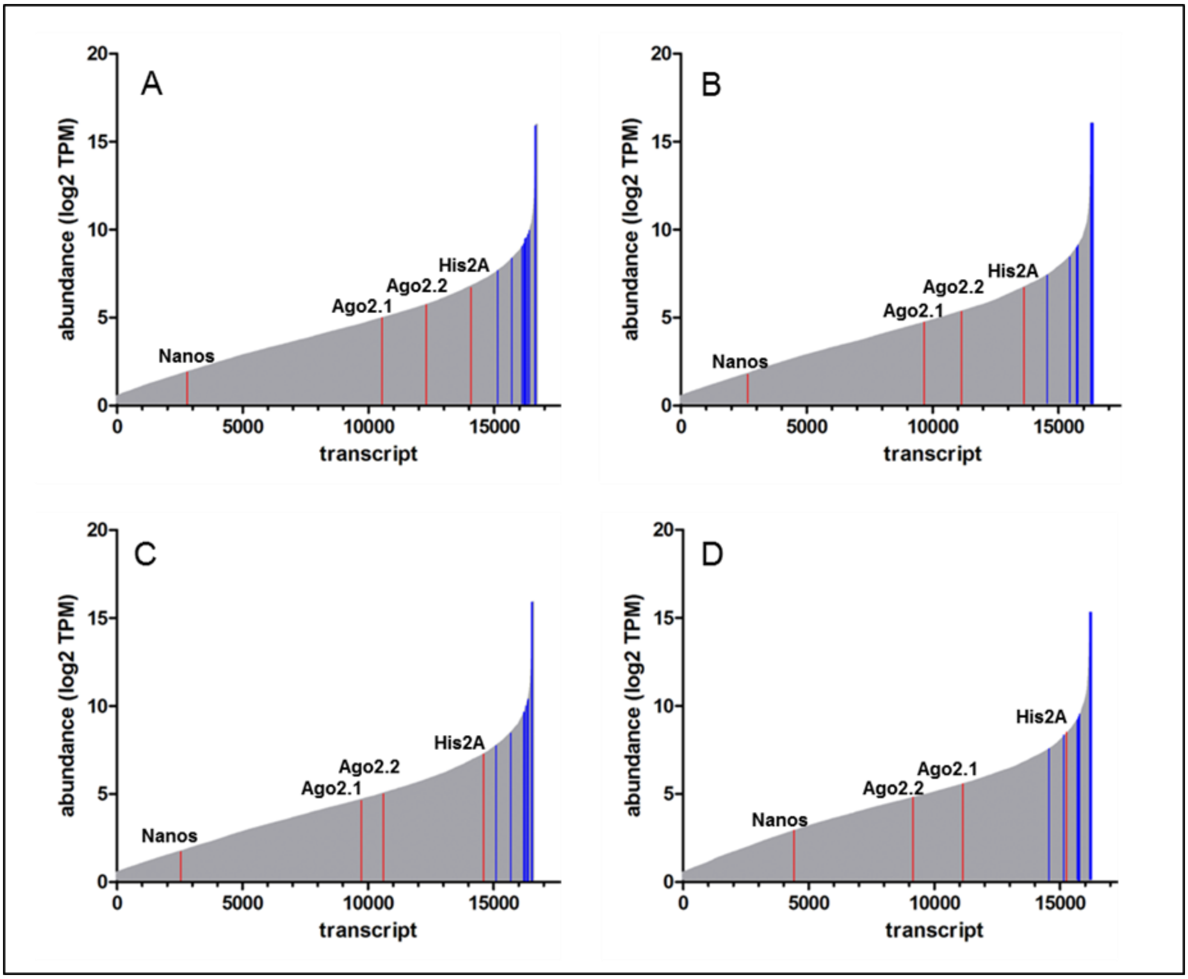

Supplemental Fig. S6: Graphical representation of the gene transcript abundance within the *F. hepatica* transcriptomes, shown as transcripts per million (TPM) on a log2 scale, from low to high transcript abundance. (A) metacercariae; (B) NEJ 1hr; (C) NEJ 3hr; (D) NEJ 24hr. The blue lines represent the genes encoding the most abundantly secreted proteins in the corresponding secretomes. The red lines represent the neoblast-associated genes.

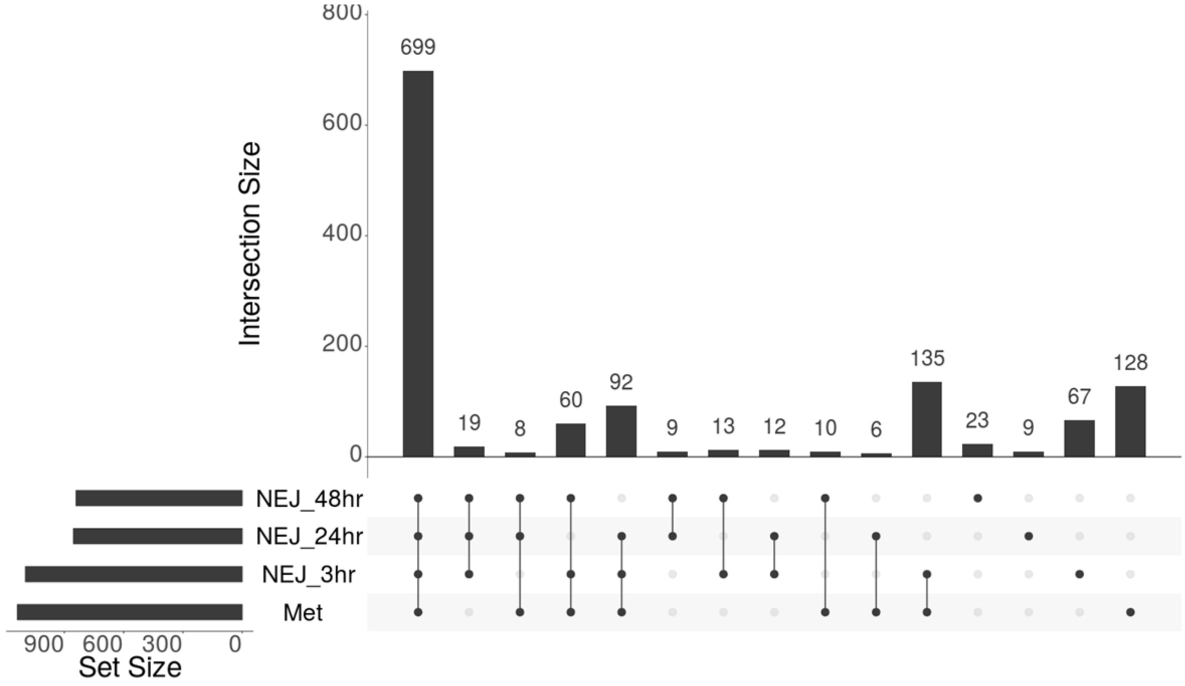

Supplemental Fig. S7: Upset plot depicting the number of proteins identified within the somatic proteome of the metacercariae, NEJ 3hr, 24hr and 48hr post-excystment lifecycle stages.
